# Quantitative genetics of photosynthetic trait variation in maize

**DOI:** 10.1093/jxb/eraf198

**Published:** 2025-05-14

**Authors:** Waqar Ali, Marcin Grzybowski, J Vladimir Torres-Rodríguez, Fangyi Li, Nikee Shrestha, Ramesh Kanna Mathivanan, Gabriel de Bernardeaux, Khang Hoang, Ravi V Mural, Rebecca L Roston, James C Schnable, Seema Sahay

**Affiliations:** Department of Agronomy and Horticulture, University of Nebraska-Lincoln, Lincoln, NE, USA; Center for Plant Science Innovation, University of Nebraska-Lincoln, Lincoln, NE, USA; Department of Agronomy and Horticulture, University of Nebraska-Lincoln, Lincoln, NE, USA; Center for Plant Science Innovation, University of Nebraska-Lincoln, Lincoln, NE, USA; Faculty of Biology, University of Warsaw, 02-096 Warsaw, Poland; Department of Agronomy and Horticulture, University of Nebraska-Lincoln, Lincoln, NE, USA; Center for Plant Science Innovation, University of Nebraska-Lincoln, Lincoln, NE, USA; Department of Agronomy and Horticulture, University of Nebraska-Lincoln, Lincoln, NE, USA; Center for Plant Science Innovation, University of Nebraska-Lincoln, Lincoln, NE, USA; Department of Biochemistry, University of Nebraska-Lincoln, Lincoln, NE, USA; Department of Agronomy and Horticulture, University of Nebraska-Lincoln, Lincoln, NE, USA; Center for Plant Science Innovation, University of Nebraska-Lincoln, Lincoln, NE, USA; Department of Horticultural Sciences, Texas A and M University, College Station, TX, USA; Center for Plant Science Innovation, University of Nebraska-Lincoln, Lincoln, NE, USA; Department of Biochemistry, University of Nebraska-Lincoln, Lincoln, NE, USA; Center for Plant Science Innovation, University of Nebraska-Lincoln, Lincoln, NE, USA; Department of Agronomy, Horticulture & Plant Science, Brookings, SD 57007, USA; Center for Plant Science Innovation, University of Nebraska-Lincoln, Lincoln, NE, USA; Department of Biochemistry, University of Nebraska-Lincoln, Lincoln, NE, USA; Department of Agronomy and Horticulture, University of Nebraska-Lincoln, Lincoln, NE, USA; Center for Plant Science Innovation, University of Nebraska-Lincoln, Lincoln, NE, USA; Center for Plant Science Innovation, University of Nebraska-Lincoln, Lincoln, NE, USA; Department of Biochemistry, University of Nebraska-Lincoln, Lincoln, NE, USA; University of Cambridge, UK

**Keywords:** *Arabidopsis thaliana*, maize, natural variation, photosynthetic traits, quantitative genetics, spatial variation

## Abstract

Natural genetic variation in photosynthesis-related traits can help both to identify genes involved in regulating photosynthetic processes and to develop crops with improved productivity and photosynthetic efficiency. However, rapidly fluctuating environmental parameters create challenges for measuring photosynthetic parameters in large populations under field conditions. We measured chlorophyll fluorescence and absorbance-based photosynthetic traits in a maize diversity panel in the field using an experimental design that allowed us to estimate and control multiple confounding factors. Controlling the impact of day of measurement and light intensity as well as patterns of two-dimensional spatial variation in the field increased heritability for 11 out of 14 traits measured. We were able to identify high-confidence genome-wide association study (GWAS) signals associated with variation in four spatially corrected traits (the quantum yield of PSII, non-photochemical quenching, redox state of Q_A_, and relative chlorophyll content). Insertion alleles for Arabidopsis orthologs of three candidate genes exhibited phenotypes consistent with our GWAS results. Collectively these results illustrate the potential of applying best practices from quantitative genetics research to address outstanding questions in plant physiology and to understand natural variation in photosynthesis.

## Teaser

Our GWAS identified high confidence signals associated with variation in relative chlorophyll, ΦPSII, ΦNPQ, and qL. Insertion alleles of Arabidopsis orthologs of maize candidate genes exhibited photosynthesis-related phenotypes consistent with the GWAS results.

## Introduction

Ongoing and forecasted climate changes are placing a growing strain on global food security and make ongoing increases in crop resilience and productivity essential. Improvements in photosynthetic efficiency have the potential to significantly increase yields (Long *et al*., 2015). Photosynthesis, the biological process of converting light energy and atmospheric CO_2_ into chemical energy and organic compounds, is a vital process for the sustainability of food production systems in the world. A better understanding of the photosynthetic processes may help to identify target traits and target genes for improving inherent photosynthetic efficiency to further enhance productivity without expanding land use, increasing water consumption, or requiring further growth in nitrogen fertilizer applications. A number of proof-of-concept studies have demonstrated improvements in photosynthetic efficiency and productivity in crop plants through transgenic approaches (Kromdijk *et al*., 2016; Driever *et al*., 2017; López-Calcagno *et al*., 2019; De Souza *et al*., 2022). Genetic engineering-based approaches that have demonstrated potential to increase photosynthetic productivity include the manipulation of various photosynthetic traits such as non-photochemical quenching (NPQ), mesophyll conductance, Rubisco activity, and sedoheptulose-bisphosphatase via overexpression to increase photoprotection, water use efficiency, CO_2_ fixation, and stomatal regulation, which led to improve photosynthetic efficiency and yield in tobacco, Arabidopsis, and rice (Kromdijk *et al*., 2016; Simkin *et al*., 2017; Hubbart *et al*., 2018; South *et al*., 2019). However, it is important to note that not all transgenic efforts to engineer photosynthesis to improve productivity have been successful (Garcia-Molina and Leister, 2020; Lehretz *et al*., 2022).

It may also be possible to exploit natural genetic variation for photosynthetic parameters in crop plants either to identify targets of genetic engineering in order to improve photosynthetic efficiency (Gu *et al*., 2014; Basu *et al*., 2019; Faralli and Lawson, 2020; Acevedo-Siaca *et al*., 2021; Meena *et al*., 2021; Sahay *et al*., 2023, 2024a) or to optimize photosynthesis capacity via marker-assisted selection or genomic selection (van Bezouw *et al*., 2019; Theeuwen *et al*., 2022). It has been suggested that exploiting variations in multiple photosynthetic traits and aggregating them could produce synergistic impacts on crop yields (Sinclair *et al*., 2019; Araus *et al*., 2021). However, as photosynthetic performance varies in response to light intensity, temperature, access to water, and other stresses, as well as in response to diurnal and circadian cycles, quantifying the impacts of natural variants for the large populations used for quantitative genetic investigation or crop improvement can be challenging under field conditions.

Rapid fluorescence-based approaches to measure photosynthetic traits have been employed in high-throughput forward genetic screens under controlled conditions to identify a number of the genes encoding core components of the photosynthetic regulatory apparatus in algae and the model plant *Arabidopsis thaliana* (Niyogi *et al*., 1997, 1998; Baker, 2008). More recently, several field-portable technologies have become available for rapidly estimating photosynthesis-related parameters using a combination of fluorescence- and absorbance-based technologies. Previous efforts to use these technologies to identify genes controlling natural variation in photosynthetic parameters have been limited by the low heritability of photosynthetic traits measured under field conditions (Dramadri *et al*., 2021; Liu *et al*., 2023). However, these previous attempts did not directly control for the impact of changes in environmental factors during data collection on the traits of interest nor did they model and control for 2D spatial variation within individual field experiments.

Here, we evaluated the potential of incorporating statistical controls for the impact of fluctuating environmental conditions to enable the identification of genes controlling variation in photosynthesis-related traits under field conditions using low-cost, field-portable tools. We collected data from a large replicated maize diversity panel in the field employing an experimental design that allowed us to separately estimate the impact of multiple confounding environmental factors. Consistent with previous reports, the photosynthesis-related traits which we scored largely exhibited extremely low heritabilities (e.g. the proportion of total variance attributable to differences between genotypes) in our initial model, but controlling for confounding environmental factors allowed many more traits to reach levels of heritability suitable for directed breeding efforts via genomic prediction or for gene discovery via genome-wide association studies (GWAS). We identified well-supported trait-associated markers in GWAS for four of the 14 traits initially profiled. In three cases, insertion alleles of the Arabidopsis orthologs of maize candidate genes also exhibited photosynthesis-related phenotypes consistent with the maize GWAS results. However, further in-depth characterization efforts will be necessary to confirm the roles of the candidate genes identified in determining variation in photosynthetic traits under field conditions.

## Materials and methods

### Maize association panel field experiment

A set of 752 maize (*Zea mays* L.) genotypes were grown in two randomized blocks of 840 plots for a total of 1680 plots, consisting of one entry of each unique genotype per block, with remaining plots filled with a single repeated check, at the University of Nebraska–Lincoln's Havelock Farm (40.852N, 96.616W). These lines were drawn from the Wisconsin Diversity Panel which consists of inbred lines selected to maximize the representation of the genetic diversity present in maize with the constraint of selecting only lines which can successfully grow and complete their life cycle in the northern temperate USA. The panel includes representation of the three major heterotic groups present in North American dent corn breeding (stiff stalk, iodent, and non-stiff stalk) from both public sector and private breeding programs, as well as a number of more diverse entries including sweet corns, popcorns, and germplasm carrying significant introgressions of tropical material (Mazaheri *et al*., 2019). A list of the specific genotypes employed in our study is provided in Supplementary Data File S1 at https://figshare.com/s/8af0d240a1f39e6b6885. During the experiment period, the field experienced daily maximum temperatures ranging from 18 °C to 36 °C, daily minimum temperatures ranging from 11 °C to 27 °C, and daily average temperatures ranging from 15 °C to 31 °C. The daily average humidity between planting and data collection ranged from 44% to 89%, the daily average wind speed from 1 m s^–1^ to 10 m s^–1^, and total precipitation was 313 mm. Each plot consisted of two parallel rows of plants from a single genotype with 30 in (0.76 m) separating the two rows. The rows ran from east to west and individual plots were positioned in a 60 East–West × 28 North–South grid. The experimental field was surrounded by an additional border of plots planted with a single uniform genotype to mitigate edge effects (Supplementary Fig. S1). Final plant density varied due to differences in germination and plant survival rates, as well as the removal of any off-type plants observed prior to sampling. The seeds were sown using a four row Almaco plot planter on the 6 May 2020. Nitrogen fertilizer was supplied via a single urea ammonium nitrate application prior to planting. Weeds were controlled by a pre-emergence application of atrazine (Syngenta) according to the manufacturer's recommendations, followed by manual weed removal throughout the growing season. No irrigation was provided prior to or during the growing season. The layout and the details of the field set-up have been previously described (Mural *et al*., 2022; Sun *et al*., 2022; Sahay *et al*., 2023).

### Scoring fluorescence- and absorbance-based photosynthesis-related traits

Fluorescence- and absorbance-based photosynthetic-related traits were scored using a set of six field-portable spectrophotometers (MultiSpeQ V2.0; PhotosynQ, East Lansing, MI, USA) (Kuhlgert *et al*., 2016) between 09.00 h and 14.30 h on 23 July (1397 plots phenotyped), 24 July (1665 plots phenotyped), 25 July (280 plots phenotyped), and 28 July (1679 plots phenotyped). All the measurements were performed using the pulse-amplitude-modulation (PAM) method as implemented using the Photosynthesis Rides 2.0 protocol provided by the manufacturer. The 14 traits quantified and analyzed as part of this study were chlorophyll SPAD (relative chlorophyll content), light-adapted maximum efficiency of PSII in the light (*F*_v_'/*F*_m_'), redox state of quinone A (Q_A_; qL), total electrochromatic shift (ECS_T_), proton conductivity (gH^+^), steady-state proton flux (vH^+^), transient values of theoretical NPQ (NPQ_T_), quantum yields of PSII (ΦPSII), NPQ (ΦNPQ), and other non-regulated energy dissipation (ΦNO), PSI active centers (PSI-ac), PSI open centers (PSI-opc), PSI oxidized centers (PSI-oxc), and PSI over-reduced centers (PSI-orc). Each plot, excluding those without any healthy plants present, was measured three times, with each measurement occurring on a different day to avoid confounding any potential date of sampling effect with genotype effects. For each measurement, a plant within the plot was arbitrarily selected by the sampler, avoiding edge plants when possible. All measurements were performed on a fully expanded leaf, targeting a point midway between the ligule and the leaf tip. The field-portable spectrophotometers automatically recorded the specific time, date, intensity of photosynthetically active radiation, ambient temperature, and ambient humidity data during each measurement.

### Phenotypic data processing and quality control

#### Variance partitioning

The variance contribution of different factors for each trait of interest was estimated by fitting models to explain the individual measurements of each trait of interest using the lme4 package in R (Bates *et al*., 2015). The model fit can be described by the equation


(1) y=μ+g+r+c+d+l+ϵ

where μ is the overall mean, *g* is the random effect of ith genotype, *r* is the random effect of the row, *c* is the random effect of the column, *d* is the random effect of the day, *l* is the random effect of light intensity, and ϵ is the residual error. Final proportion values were obtained by dividing the variance attributed to each factor for a given trait by the total observed variance for that trait.

### Heritability

Initial broad-sense heritability estimates were generated using replicated plot-level average values for each trait. In most cases, each genotype was represented by two separate plots. These plot-level averages were calculated after dropping individual measurements with extreme values outside of the expected range for each trait (see cut-offs in Supplementary Fig. S2 and Supplementary Table S1). The proportion of variance attributable to genotypes was estimated in a linear model fitting genotype as random. The linear model is given as


(2) $$Y=\mu +\delta_{i}+\epsilon_{i,j}$$

where *Y* is the response variable, μ is the overall mean, δ*_i_* is the random effect of the *i*th genotype, and $\epsilon_{i,j}$ is the residual error for the *j*th plant of the *i*th genotype. Variance components were extracted, and broad-sense heritability was estimated using the following formula:


(3) $$H=\frac{\sigma_{G}^{2}}{\sigma_{G}^{2}+\frac{1}{n}\sigma_{R}^{2}}$$

where $\sigma_{G}^{2}\;$ is the genotypic variance, $\sigma_{R}^{2}$ is the residual variance, and *n* is the number of replications per genotype (two in our experimental design).

Final heritability estimates were generated using corrected plot-level values (calculated for each plot based on up to three independent measurements per plot) generated in SpATS rather than the simple plot-level averages employed for the initial heritability estimates. Corrected plot-level values were generated in SpATS v1.0-18 (Rodríguez-Álvarez *et al*., 2018) fitting light intensity and ambient temperature as fixed effects, plotID and day as random effects, and modeling 2D spatial effects on each phenotype using 15 column knots and 31 row knots. The SpATS model employed to generate corrected plot-level values is described by the following equation:


(4) $$y=X\beta +Z_{s}\mu_{s}+Z_{g}g+f(x,z)+\epsilon$$

Where *y* is the response variable, *X*β denotes the fixed effects, *Z*_s_μ_s_ denotes the random effects, *Z*_g_g denotes the plotID taken as random, *f*(*x*,*z*) denotes the smooth surface function (modeling spatial trends), and ϵ is the residual error.

After correcting for spatial effects and confounding factors in SpATS, the corrected heritability estimates were generated using the same approach as the initial heritability estimates. The variance components were extracted by fitting genotypes as random within a linear model implemented in lme4, variance components were extracted, and heritability was estimated using Equation 3.

Best linear unbiased estimates (BLUEs) used for GWAS were generated from individual measurement data points, typically including six total observations per genotype collected across two plots, although in some cases this was less as the result of the removal of measurements with extreme values. These plot-level data points (values) were corrected using the SpATS package fitting genotype as a fixed effect, light intensity and ambient temperature as fixed effects, day as a random effect, and modeling 2D spatial effects for each phenotype using 15 column knots and 31 row knots (Supplementary Fig. S3). This model can be described by Equation 4, with the modification that here *Z*_g_g denotes the genotype taken as fixed.

### Genome-wide association studies

GWAS were conducted using either the FarmCPU algorithm (photosynthesis-related traits) or the MLM algorithm [expression quantitative trait locus (eQTL) analysis], each as implemented in the rMVP package (version, 1.0.8) (Liu *et al*., 2016; Yin *et al*., 2021) and each including three principal components (PCs) calculated from genetic marker data as additional covariates. These three PCs, in aggregate, explained >75% of the total variance in genetic markers in our dataset, with PC1 explaining 62.3% of the variance, PC2 explaining 7.9%, and PC3 explaining 6.8%. While the FarmCPU model used mapping of photosynthesis-related trait controls for kinship via an alternative method as part of the association testing itself [specifically it calculates kinship derived from pseudo quantitative trait nucleotides (QTNs)], the MLM-based GWAS employed for the eQTL analysis employed an externally calculated kinship matrix to control for the confounding effects of relatedness among individuals in a population. The resequencing-based marker set generated in Grzybowski *et al*. (2023) was subset to only those biallelic markers which exceeded a minor allele frequency of 0.05 considering only those individuals represented in this study and excluding markers where >20% of genotypes were classified as heterozygous, resulting in a set of 11.8 million genetic markers. The effective number of independent markers represented by this dataset was estimated to be 4.7 million via GEC 0.2 (Li *et al*., 2012). For each trait, the FarmCPU GWAS analysis was conducted 100 times, with 10% of trait values masked for each iteration and a *P* threshold setting of 1.06 × 10^−8^, calculated based on the Bonferroni correction applied to an initial *P*-value of 0.05 and the estimated number of independent markers. Markers were considered to be significant in a given interaction if they exceeded a *P*-value threshold of 1.06 × 10^−8^. Resampling model inclusion probabilities (RMIPs) were calculated by dividing the number of iterations in which that marker was significantly associated with a given trait (*P* < 1.06 × 10^−8^) by the total number of iterations run (100). Linkage disequilibrium (LD) between markers in mapping intervals was estimated using PLINK 1.9 (Purcell *et al*., 2007).

### Chlorophyll ground truth data

Absolute chlorophyll content was scored separately from the measurements described above, with data collected from a single leaf of a single plant from each of 318 plots using a handheld chlorophyll meter (MC-100, Apogee Instruments, Inc., Logan, UT, USA). Absolute chlorophyll measurements were collected on 9 d spanning a 13 d sampling period between 8 July and 20 July 2020 (see Tross *et al*., 2024).

### Expression quantitative trait locus analysis

The eQTL results presented in this study were obtained using gene expression data of 693 genotypes profiled using mRNA-seq of RNA samples extracted from mature leaf tissue collected in a 2 h period on 8 July 2020 (Torres-Rodríguez *et al*., 2024). QTL mapping was performed using the mixed linear method as implemented within the rMVP package (1.0.8) in R (Price *et al*., 2006; Yin *et al*., 2021) and the same set of 11.8 million segregating markers employed for GWAS above. Associations between markers and gene expression were considered significant if they exceeded a *P*-value threshold of 1.06 × 10^−8^.

### Characterization of Arabidopsis mutants

T-DNA insertion lines were tested for three genes: SALK_007055, carrying an insertion in AT1G10500 (*ATCPISCA* or *Chloroplast-localized ISCA-like protein*); SALK_080503, carrying an insertion in AT2G26660 (*ATSPX2* or *SPX domain gene 2*); and SALK_047115, carrying an insertion in AT5G42520 (*ATBPC6* for *Basic Pentacysteine 6*). Homozygosity of each T-DNA insertion within individual plants from each stock was confirmed by PCR, using the T-DNA-specific primer LBb1.3 along with gene-specific left and right primers designed through the Salk T-DNA primer design tool (http://signal.Salk.edu/tdnaprimers.2.html) and detailed in Supplementary Table S2.

Seeds for all T-DNA insertional mutants and their wild-type control (Columbia ecotype, Col-0, CS6000) were obtained from the Arabidopsis Biological Resource Center (ABRC) at Ohio State University (Alonso *et al*., 2003). Seeds were stratified for 4 d at 4 °C in the dark, then sown in 3.5 in × 3.5 in (8.89×8.89 cm) pots (SQN03500B66, Hummert International, Earth City, MO, USA) filled with soil-less potting mix (1220338; BM2 Germination and Propagation Mix; Berger, SaintModeste, Canada). Pots were placed in trays (6569630; Hummert International) filled with 2 cm of water at the bottom and covered with a clear plastic dome (65696400; Hummert International) until germination. The trays were placed in a reach-in growth chamber (AR-66L2; Percival, Perry, IA, USA) at 21 °C day/18 °C night with a 10 h light/14 h dark photoperiod (200 μmol m^−2^ s^−1^) and 60% relative humidity. After germination, the domes were removed, and seedlings were allowed to grow for 10 d. Then seedlings were thinned to one plant per pot. Plants were watered and repositioned at random locations in the chamber three times a week.

Relative chlorophyll, ΦPSII, and ΦNPQ were measured using MultispeQs (PhotosynQ, East Lansing, MI, USA; Kuhlgert *et al*., 2016) with the same protocol (Photosynthesis Rides 2.0) employed for field measurement. The initial set of low-light measurements were taken on 4-week-old plants under low-light conditions (LL; 200 μmol m^−2^ s^−1^) in the same reach-in chambers where they were grown under controlled condition as described above. High-light measurements were collected from the same plants after the plants were moved to a high-light treatment (HL; 550 μmol m^−2^ s^−1^ at 24 °C/22 °C day/night with an 8 h day/16 h night) in a walk-in growth chamber (Conviron model GR48, Controlled Environments, Manitoba, Canada) for 24 h. The measurements were performed on fully expanded leaves from the middle of the rosette of the plant with data collection occurring between 10:30 h and 12.30 h.

### Statistical analysis

Statistical analyses of photosynthetic parameter measurements in Arabidopsis mutants were performed using R software v.4.3.2. As all measurements were collected in <2 h from inside a controlled environment (growth chamber), we did not correct for confounding factors. Given the simplicity of this comparison, we were able to employ an unpaired two-tailed *t*-test and did not employ lme4.

## Results

### Repeatability of field-scored photosynthetic traits is enhanced by removing outliers and controlling for spatial variation

**Fig. 1.**
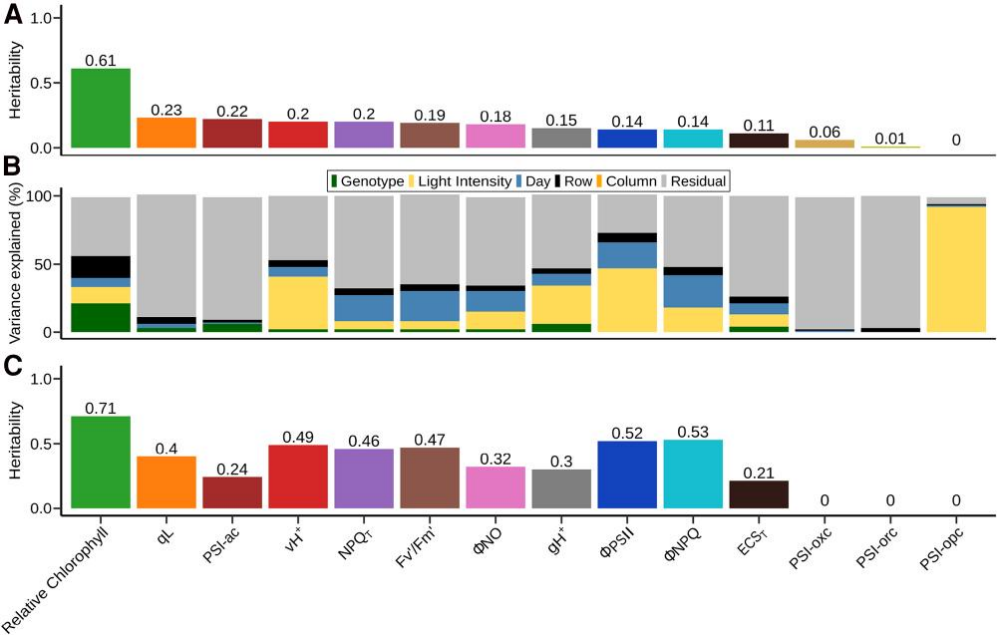
Factors explaining variation in measured values for photosynthesis-related traits under field conditions. (A) Estimated genotype-level heritability across two replicated plots in the same field for plot-level averages (*n* = 2), uncorrected for environmental or spatial confounders, of 14 traits scored in the field using MultiSpeQs. (B) Estimated proportion of variance in individual measurements (*n* = 6) of the same 14 traits attributable to genotype, light intensity recorded at the time of collection, day on which the measurement was taken, and row and column position within the field. (C) Estimated genotype-level heritability across two replicated plots (*n* = 2) in the same field for plot-level trait estimates generated after correcting for the effects of light intensity and day as well as 2D spatial variation throughout the field using SpATS.

Considering simple plot-level averages for the three independent measurements collected per plot, all photosynthesis-related traits assayed in this experiment, with one exception, exhibited heritability of <0.25 (Fig. 1A). The sole exception was relative chlorophyll content, with an estimated heritability of 0.61. With the exception of PSI-ac, all PSI-related traits exhibited heritabilities <0.1. The heritabilities for photosynthesis-related traits were substantially lower than those observed for whole-plant phenotypes measured in the same field experiment. The mean and median heritability of 28 manually measured plant phenotypes collected from the same field (Mural *et al*., 2022) were 0.75 and 0.85, respectively.

The distribution of raw values (i.e. without correction for spatial variation or other confounding factors) recorded for many of the photosynthesis-related traits were significantly different across the 4 d over which data collection occurred. Across a wide enough range of conditions, many photosynthetic parameters exhibit non-linear responses to change in light intensity or other environmental factors. However, we found that, for the range of environmental values observed during our field data collection, the relationship between environmental and photosynthetic parameters could be approximated with a linear model, simplifying model fitting, although in several cases modest non-linear effects are also observable (Supplementary Fig. S4). Day of collection explained 7% of the variation in relative chlorophyll, 19% in ΦPSII and 24% in ΦNPQ of total variance (Fig. 1B; Supplementary Table S3). Light intensity varied substantially over the time period during which data were collected, with several prolonged periods of lower intensity light resulting from clouds obscuring the sun (Supplementary Fig. S5). Variation in light intensity at the time of collection also explained a percentage of variance ranging from 12% to 47% of the total variation for relative chlorophyll, ECS_T_, gH^+^, vH^+^, and ΦPSII, and explaining 92% of the variation in PSI-opc, a trait with essentially no heritability between replicates of the same genotype in different parts of the field (Fig. 1B; Supplementary Table S3). Sixteen percent of the variance in measured relative chlorophyll content was explainable by differences between different rows in the field. It should be noted that this is likely to be an underestimate of the total variation in these traits explained by within-field spatial variation as many factors with the potential to influence plant phenotypes, such as nutrient availability, soil type, soil moisture, and elevation, will vary along gradients not easily captured by either row or column variables.

We fit a model to control for a number of covariates (see the Materials and methods and Equation 4) as well as 2D spatial variation throughout the field, a method that yields superior correction for within-field variation relative to fitting row and column effects (1D spatial variation) (Rodríguez-Álvarez *et al*., 2018). The heritability of corrected plot-level estimates was greater than the heritability of uncorrected estimates in 11 out of 14 phenotypes (Fig. 1C). A similar pattern was observed using heritability estimated by the R package SpATS fitting a model directly to the ∼5000 individual measurements collected with models that incorporated corrections for spatial variation and covariates resulting in higher estimated heritability than models which did not include these factors (Supplementary Table S4). ΦPSII and ΦNPQ, estimates of the proportions of light captured by PSII which are employed for productive photochemistry and dissipated via photoprotection mechanisms, respectively, showed the largest increases in heritability. The heritability of ΦPSII increased from 0.14 when using simple average values to 0.52 when correcting for spatial variation and confounding environmental factors. Similarly, the heritability of ΦNPQ increased from 0.14 to 0.53. Traits related to PSI exhibited the smallest increases in heritability and included the only traits where correction for confounding factors either failed to increase heritability (PS1-opc) or decreased heritability (PS1-orc).

### Genetic loci linked to variations in photosynthesis-related traits

**Fig. 2.**
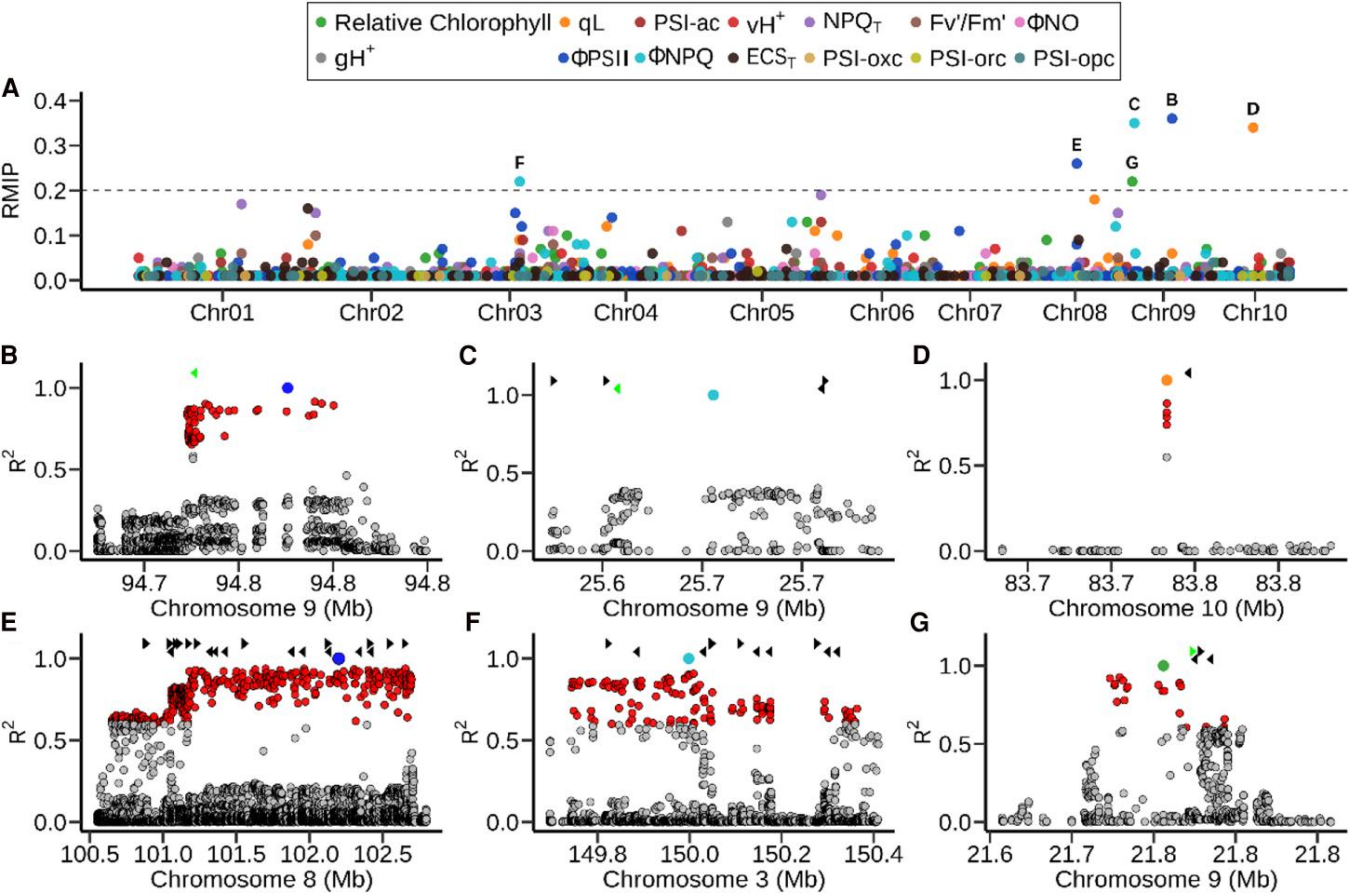
Genetic markers in the maize genome significantly associated with variation in photosynthetic traits under field conditions. (A) Statistical support and position on the maize genome for individual genetic markers associated with variation in 14 photosynthetic traits scored for maize genotypes from the Wisconsin Diversity panel under field conditions in 2020. Position on the *x*-axis indicates the physical position of the marker on the B73 RefGen V5 genome assembly. Position on the *y*-axis indicates the proportion of 100 iterations of FarmCPU GWAS where the marker was significantly associated with the given trait. Dashed line indicates an RMIP threshold of >0.2. Letters indicate the figure panel where further detail is provided for individual markers identified above this threshold. (B) Region containing markers in elevated linkage disequilibrium (LD) with a genetic marker associated with variation in ΦPSII located at position 94 775 951 on chromosome 9. Circles indicate the positions (*x*-axis) and LD with the trait-associated marker (*y*-axis) for other genetic markers in this interval. To capture the context of LD surrounding the interval of interest, the range shown begins ∼4 kb before the first marker in LD >0.6 with the trait-associated marker (red circles) and extends ∼4 kb beyond the last marker in LD with the trait-associated marker. Triangles indicate the positions and strands of annotated gene models within this interval. (C) Region containing markers in elevated LD with a genetic marker associated with variation in ΦNPQ located at position 25 683 346 on chromosome 9. All figure elements are as defined in (B). (D) Region containing markers in elevated LD with a genetic marker associated with variation in qL located at position 83 741 616 on chromosome 10. All figure elements are as defined in (B). (E) Region containing markers in elevated LD with a genetic marker associated with variation in ΦPSII located at position 102 200 025 on chromosome 8. All figure elements are as defined in (B). (F) Region containing markers in elevated LD with a genetic marker associated with variation in ΦNPQ located at position 83 741 616 on chromosome 3. All figure elements are as defined in (B). (G) Region containing markers in elevated LD with a genetic marker associated with variation in relative chlorophyll located at position 21 755 970 on chromosome 9. All figure elements are as defined in (B). In (B), (C), and (G), green triangles highlight the genes which were characterized in this study.

Genetic markers could be linked to variation in four of the 14 traits measured in this study at an RMIP (the proportion of times a marker is included in the resampling data based on iterations in the FarmCPU model) threshold of >0.2, namely relative chlorophyll content (one hit), ΦPSII (two hits), ΦNPQ (two hits), and qL (one hit) (Fig. 2A; Table 1;  Supplementary Data File S2 at https://figshare.com/s/8af0d240a1f39e6b6885). The FarmCPU model handles single nucleotide polymorphisms (SNPs) in an iterative manner, adding genetic markers with significant effects as covariates in the model. As a result, genomic intervals containing a genetic variant influencing the trait of interest will typically be represented by only one genetic marker in the results of FarmCPU-based GWAS, rather than all genetic markers in LD with the variant of interest as is typically seen in the results of GWAS conducted using other approaches. These four traits included the three traits with the highest heritability among those examined in this study after correcting for spatial variation and other confounding factors. A parallel study conducted including another statistical model which also sought to account for device-to-device variation in measurement, a rough proxy for identity of the person making measurements and selecting which plants to measure, provided roughly equivalent results (Supplementary Fig. S6). Of the six trait-associated markers which exceeded an RMIP threshold of 0.2 in our analysis, four also met or exceeded an RMIP of 0.2, one approached but did not meet that threshold, and one exhibited a substantially reduced signal strength (Supplementary Table S5). When GWAS was conducted using BLUEs calculated without the inclusion of ambient temperature as a covariate, all trait-associated markers exhibited substantially reduced significance (Supplementary Table S5).

**Table 1.** Genomic regions and trait association summary from genome-wide association studies

CHR	POS	Trait	RMIP	Associated genomic interval	No. of genes	Other associations
Chr9	94775951	ΦPSII	0.36	94 725 951–94 825 951	1	ΦNPQ, 0.02
Chr9	25683346	ΦNPQ	0.35	25 633 346–25 733 346	5	
Chr10	83741616	qL	0.34	83 691 616–83 791 616	1	*F*_v_'/*F*_m_', 0.01
Chr8	102200025	ΦPSII	0.26	102 150 025–102 250 025	22	
Chr3	149998190	ΦNPQ	0.22	149 948 190–150 048 190	12	
Chr9	21755970	Relative chlorophyll	0.22	21 705 970–21 805 970	4	

Between one and 22 annotated gene models were considered plausible candidate genes, defined as those located no more than 50 kb from either the trait-associated marker or other markers in LD (*R*^2^) >0.6 with the trait-associated marker (Table 1). The signal on chromosome 9 associated with variation in ΦPSII was located within the gene body of Zm00001eb386270 (Fig. 2B; Supplementary Table S6), encoding an SPX domain-containing membrane protein, which has been referred to as either *SPX6* (Xiao *et al.*, 2021) or *SPX1* (Luo *et al*., 2024). Below, to avoid confusion with the Arabidopsis orthologs, also assigned numerical SPX designations, we will refer to this gene as *ZmSPX1*. The signal on chromosome 9 associated with variation in ΦNPQ was in an interval containing five annotated genes including a peptidyl-prolyl *cis-trans* isomerase (Zm00001eb378260) and *bbr4* (Zm00001eb378270), a member of the BPC plant-specific transcription factor family (Fig. 2C; Supplementary Table S6). Analysis of both transcription factor-binding site enrichment and co-expression networks suggests that *ZmBBR4* represses the expression of many photosynthesis-related genes and contributes to both diurnal regulation of photosynthetic genes and the differentiation of expression between mesophyll and bundle sheath cells (Borba *et al*., 2023). The signal on chromosome 10 associated with variation in qL specifically tagged an MYB transcription factor (*ZmMYB26*; Zm00001eb416530) annotated as playing a role in leaf senescence (Fig. 2D; Supplementary Table S6). The signal on chromosome 8 associated with variation in ΦPSII was located in a region of elevated LD including 22 annotated genes (Fig. 2E; Supplementary Table S6). The signal on chromosome 3 associated with variation in ΦNPQ was also located in a region of elevated LD with 12 annotated gene models located within the plausible interval around the trait-associated SNP (Fig. 2F; Supplementary Table S6). The final marker–trait association which exceeded an RMIP threshold of 0.2 was a marker located at ∼22 Mb on chromosome 9 associated with variation in relative chlorophyll content. The LD window surrounding this gene included a cluster of four annotated gene models (Fig. 2G; Supplementary Table S6).

**Fig. 3.**
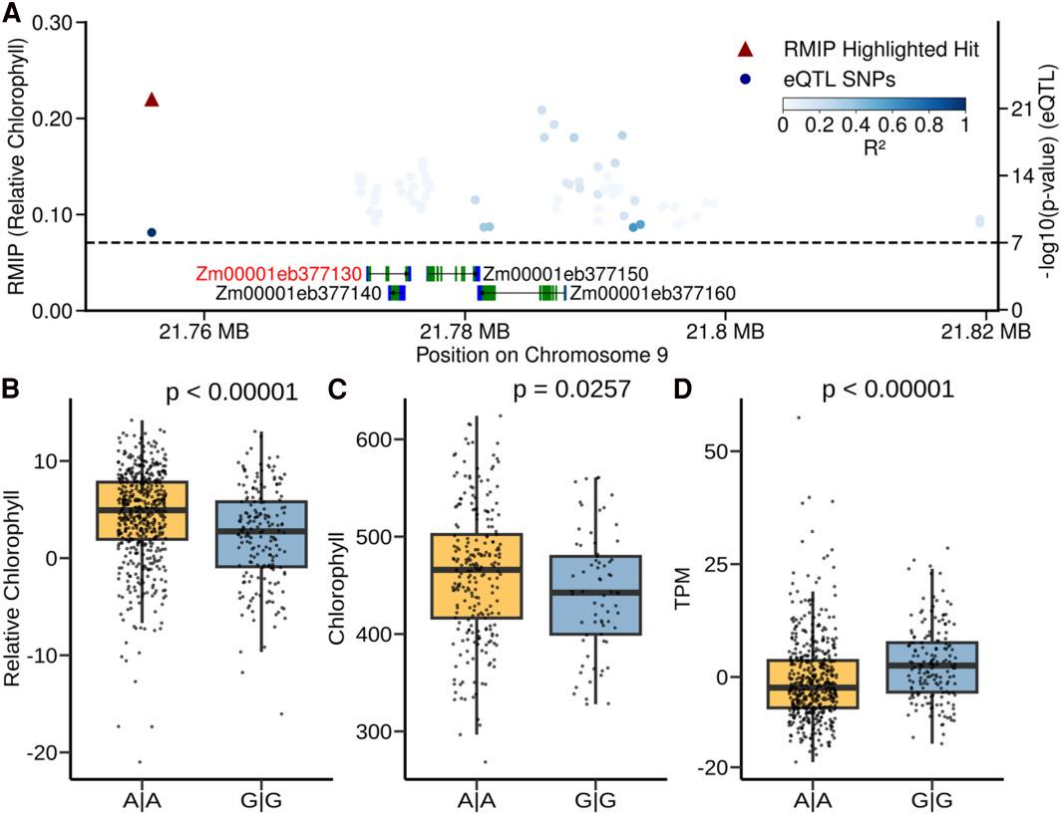
A genetic marker associated with variation in relative chlorophyll content is also associated with variation in absolute chlorophyll content and the expression of a nearby gene encoding a plastid-localized protein. (A) Region containing both the genetic marker associated with relative chlorophyll content (Chr09:21 755 970, red triangle) and the four genes contained within the linkage disequilibrium (LD)-defined interval surrounding this marker. Black arrows indicate the strand of genes, green boxes the positions of protein-coding exons, and blue boxes the positions of 5'- and 3'-untranslated regions (UTRs). Blue circles indicate the positions (*x*-axis) of genetic markers significantly associated with variation in the expression of Zm00001eb377130 and statistical significance of that association (*y*-axis). Color of circles indicates the degree of LD between markers associated with variation in gene expression and Chr09:21 755 970. (B) Difference in relative chlorophyll content between maize genotypes homozygous for the major (A|A) (*n* = 519) and minor (G|G) alleles (*n* = 183) of Chr09:21 755 970. The *P*-value shown was calculated via two-sample *t*-test implemented in R. (C) Difference in absolute chlorophyll content for a subset of maize genotypes scored using a handheld chlorophyll meter taken from Tross *et al*. (2024). Genotypes homozygous for the major (A|A) alleles (*n* = 178). Genotypes homozygous for the minor (G|G) allele (*n* = 66). (D) Difference in expression of Zm00001eb377130 in mature leaf tissue between maize genotypes homozygous for the major (A|A) alleles (*n* = 480) and minor (G|G) alleles (*n* = 171) of Chr09:21 755 970. The *P*-value shown was calculated via a unpaired two-tailed *t*-test as implemented in R.

The gene model, Zm00001eb377130, closest to the trait-associated marker for relative chlorophyll on chromosome 9 encodes a chloroplast-localized protein involved in iron–sulfur (Fe–S) metabolism (Fig. 3A). The SNP associated with variation in relative chlorophyll content assessed using the method employed in this study (Fig. 3B) was also significantly predictive of absolute chlorophyll measured at a different time point for a subset of plots using an independent methodology (see the Materials and methods) (Fig. 3C). The expression of Zm00001eb377130 in mature leaf tissue is associated with a large-effect *cis*-eQTL, with the genetic markers most significantly associated with the gene located immediately downstream of the annotated gene model (Fig. 3A). The genetic marker associated with variation in relative chlorophyll content is also significantly associated with variation in the expression of Zm00001eb377130 (Fig. 3D).

### Validation of genes associated with relative chlorophyll, ΦPSII, and ΦNPQ using Arabidopsis T-DNA insertional mutants

**Fig. 4.**
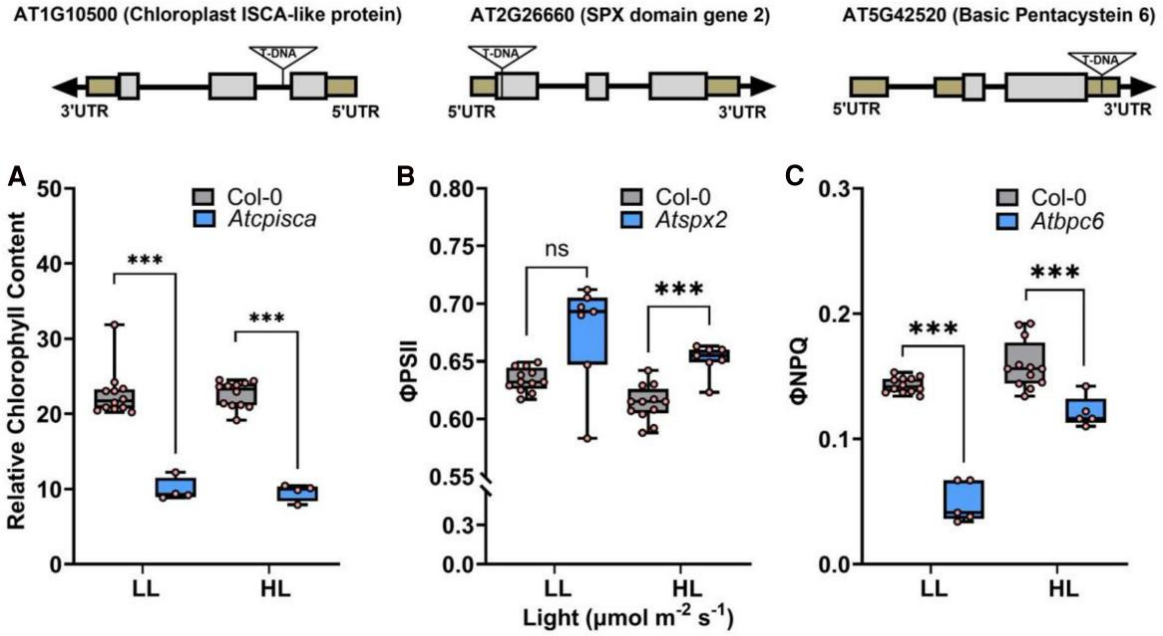
Phenotypes of insertion alleles of Arabidopsis genes homologous to maize candidate genes identified via GWAS. Plants were grown under low-light conditions (LL; 200 µmol m^−2^ s^−1^) and moved to high-light conditions (HL; 550 µmol m^−2^ s^−1^) after 4 weeks, and phenotypes were collected under both low light and high light after a 24 h acclimation to higher light intensity. (A) Difference in relative chlorophyll content between *Atcpisca* mutant (*Chloroplast-localized ISCA protein*; SALK_007055; *n* = 4) and wild-type Columbia plants (CS6000; Col-0; *n* = 12) for AT1G10500. (B) Difference in ΦPSII between *Atspx2* mutant (*SPX domain gene 2*; SALK_080503; *n* = 7) and wild-type Columbia plants (Col-0; *n* = 12) for AT2G26660. (C) Differences in ΦNPQ between *Atbpc6* mutant (*Basic Pentacysteine 6*; SALK_047115; *n* = 5) and wild-type Columbia plants (Col-0; *n* = 12) for AT5G42520. *P ≤ 0.05, **P ≤ 0.01, ***P ≤ 0.001 (unpaired, two-tailed *t*-test).

Zm00001eb377130, a candidate gene linked to variation in chlorophyll content via GWAS, and one close paralog in the maize genome, Zm00001eb270460, are co-orthologous to a single Arabidopsis gene AT1G10500 (*ATCPISCA*). Arabidopsis plants homozygous for an insertion in *Atcpisca* exhibited a substantial reduction in chlorophyll content relative to wild-type plants under both low-light (200 µmol m^−2^ s^−1^) conditions and after 24 h of high-light (550 µmol m^−2^ s^−1^) treatment (Fig. 4A). While this difference in phenotype was most significant when measured in vegetative stage plants (*P* = 0.0001), a similar pattern was observed in wild-type and *Atcpisca* mutant plants at the flowering stage, with the difference being statistically significant under low-light conditions (*P* = 0.006) but not statistically significant after high-light treatment (Supplementary Fig. S7A). Zm00001eb386270, the sole candidate gene associated with the trait-associated genetic marker for ΦPSII on chromosome 9, is orthologous to two duplicate genes in the Arabidopsis genome: AT2G26660 (*ATSPX2*) and AT5G20150 (*ATSPX1*). No significant differences were observed between Arabidopsis plants homozygous for an insertion in *Atspx2* and wild-type plants under low-light conditions, but *Atspx2* mutant plants exhibited significantly higher ΦPSII than wild-type plants after a 24 h high-light treatment at both the vegetative (*P* < 0.0001) and flowering stages (*P* = 0.03) (Fig. 4B; Supplementary Fig. S7B). Zm00001eb378270, the most proximal of the five potential candidate genes associated with the trait-associated genetic marker for ΦNPQ on chromosome 9 has a 1:1 orthologous relationship with AT5G42520 (*ATBPC6*). At the vegetative stage Arabidopsis plants homozygous for an insertion in *Atbpc6* exhibited substantially reduced ΦNPQ under both low-light conditions and high-light treatment (*P* = 0.0001), but no statistically significant differences between mutant and wild-type plants were observed at the flowering stage (Fig. 4C; Supplementary Fig. S7C).

## Discussion

Plant populations exhibit significant diversity in photosynthetic properties in addition to being highly responsive to environmental perturbations. Measurements of chlorophyll fluorescence are widely used to monitor and quantify the photosynthetic status of plants (Murchie and Lawson, 2013). Previous efforts to employ chlorophyll fluorescence to quantity genetic variation in photosynthetic parameters across large plant populations have either removed leaves or leaf disks from the field to collect measurements under controlled sets of environmental parameters (Ferguson *et al*., 2023; Sahay *et al*., 2023), or have made limited efforts to control for confounding factors via the incorporation of replicate or block effects (Dramadri *et al*., 2021; Liu *et al*., 2023).

Controlling for spatial and environmental covariates using linear models allowed us to substantially increase heritability (Fig. 1C) relative to other field studies conducted using similar approaches. Two previous studies employed the same instrument for field-based phenotyping of photosynthetic traits, an analysis of 256 common bean (*Phaseolus vulgaris* L.) accessions (Dramadri *et al*., 2021), and an analysis of 225 rice (*Oryza sativa* L.) accessions (Liu *et al*., 2023). Three of the 14 traits we analyzed in this study were also represented in both of these previous studies: ΦPSII, ΦNPQ, and ΦNO. In all three cases, the heritability we achieved after correcting for both spatial and environmental confounders substantially exceeded those reported in previous studies which were unable to control for these confounders. We observed a heritability of 0.52 for ΦPSII in maize, substantially higher than the 0.15 reported in common bean or the 0.22 reported in rice. While, in principle, this increased heritability could also be attributed to greater genetically controlled diversity for ΦPSII in the maize population we examined related to the rice and common bean populations employed in these comparison studies, the heritability we observed for ΦPSII prior to controlling for spatial and environmental confounders (0.14; Fig. 1A) is similar to those reported in these other studies. This suggests that the improvement is more likely to be attributable to our ability to capture and explain more of the non-genetic variance in measurements through the incorporation of covariates and spatial models, reducing residual variance and, thus, the size of the denominator in the heritability equation. Similar patterns are present for ΦNPQ where the reported heritabilities were 0.14 in common bean, 0.35 in rice, and we observed 0.53 in maize subsequent to spatial and environmental correction, and in ΦNO where the reported heritability was 0.08 in common bean, 0.18 in rice, and we observed 0.32 in maize subsequent to spatial and environmental correction.

Out of the six strongly supported (RMIP > 0.2) trait-associated SNPs identified as part of this study, two were located in regions of locally elevated LD which resulted in large numbers of potential causal genes, and four were located in low LD regions associated with only one or several potential causal genes (Fig. 2). We obtained homozygous insertion mutants for Arabidopsis orthologs of maize candidate genes in three of the four cases where a trait-associated SNP tagged only one or several genes. In all three cases, including one candidate gene each from GWAS conducted for relative chlorophyll, ΦPSII, and ΦNPQ, the Arabidopsis mutant phenotype was consistent with the predicted function from the maize GWAS study (Figs 2, 4). Several recent studies have reported phenotypes for sets of Arabidopsis mutants consistent with GWAS hits associated with homologs of the same genes in maize (Sahay *et al*., 2023; Scharwies *et al*., 2025). We did not observe any genetic markers significantly associated with variation in NPQ_T_. The NPQ_T_ values calculated by the MultiSpeQ assume a fixed *F*_v_/*F*_m_, although this can be corrected for, if dark-adapted *F*_v_/*F*_m_ is measured separately (Sahay *et al*., 2024b). NPQ_T_ values reported directly by the MultiSpeQ and those corrected using measured dark-adapted *F*_v_/*F*_m_ were highly correlated (*R*^2^ > 0.97) for the 20 maize genotypes measured in Sahay *at al*. (2024b) (Supplementary Fig. S8); however, it is still possible that the absence of this correction explains the lack of hits for NPQ_T_.

The gene Zm00001eb377130/AT1G10500, linked to chlorophyll content in maize via GWAS and in Arabidopsis via mutant analysis, is homologous to a well-characterized bacterial gene *ISCA* which functions in the creation of Fe–S complexes (Ayala-Castro *et al*., 2008). Fe–S complexes are involved in electron transfer reactions within chloroplasts, driving the redox processes necessary for synthesizing chlorophyll precursors (Abdel-Ghany *et al*., 2005). Specifically, Fe–S cluster proteins facilitate enzymatic activities such as those in magnesium chelatase and other steps critical to the conversion of intermediates into functional chlorophyll molecules. In Arabidopsis, the protein encoded by this gene has been shown to be chloroplast localized (Abdel-Ghany *et al*., 2005). Disruption of this gene in Arabidopsis via T-DNA insertion was associated with a substantial decline in relative chlorophyll content (Fig. 4A; Supplementary Fig. S7A), consistent with the predictions of the GWAS analysis conducted in maize.

An insertion allele of one of the two Arabidopsis orthologs of *ZmSPX1*, a gene linked to variation in ΦPSII via GWAS, exhibited a change in ΦPSII relative to wild-type plants. The Arabidopsis insertion mutant (*ATSPX2*) exhibited an increase in ΦPSII (Fig. 4B; Supplementary Fig. S7B), consistent with mutant plants utilizing a larger proportion of light energy for productive photochemistry than wild-type plants. However, this apparent increase in photosynthetic productivity is consistent with a previous report that edited alleles of *ZmSPX1* in maize exhibit increases in productivity (Luo *et al*., 2024), suggesting that *ZmSPX1* may act as a negative regulator of photosynthetic capacity. However, care should be taken in interpreting this result as many genes which apparently result in increases in yield or plant productivity fail to validate or exhibit significant phenotypic trade-offs when tested across a wider range of environments (Khaipho-Burch *et al*., 2023).

The maize candidate gene *ZmBBR4* (Zm00001eb378270) belongs to a plant-specific family of transcription factors with four members in maize and seven members in Arabidopsis. Analysis of both transcription factor-binding site enrichment and co-expression networks suggests that *ZmBBR4* represses the expression of many photosynthesis-related genes and contributes to both diurnal regulation of photosynthetic genes and the differentiation of expression between mesophyll and bundle sheath cells (Borba *et al*., 2023). Mutation of the rice ortholog of *ZmBBR4*, *OsGBP1*, was associated with greater biomass accumulation and larger seeds, with overexpression lines exhibiting reciprocal phenotypes (Gong *et al*., 2018). In Arabidopsis, single mutants in this transcription factor family had been reported to be phenotypically silent, while higher order mutants carrying loss-of-function alleles for multiple genes in this family did exhibit phenotypic effects (Monfared *et al*., 2011; Hecker *et al*., 2015). However, in our analysis, Arabidopsis plants carrying an insertion allele of *Atbpc6*, one of the Arabidopsis orthologs of *ZmBBR4*, exhibited decreases in ΦNPQ relative to wild-type plants (Fig. 4C; Supplementary Fig. S7C), a phenotype which was probably not screened for in prior efforts to characterize this gene family, demonstrating the potential of quantitative genetic analysis, even in distantly related species, to guide reverse genetics efforts in model species such as Arabidopsis.

The two GWAS hits which tagged high LD windows in the maize genome were each associated with larger numbers of candidate genes (Fig. 2E, F; Supplementary Table S4). While we did not validate any of these genes in these intervals via characterization of insertion alleles, each contains one or more genes with potential mechanistic links to variation in photosynthetic performance. The 22 annotated genes in the interval on maize chromosome 8 associated with variation in ΦPSII include Zm00001eb348280, which encodes a member of the FtsZ2 protein family, a group of proteins which play a role in chloroplast division. Two-fold overexpression of *AtFtsZ2-1* in Arabidopsis increased the number of chloroplasts whereas a 3-fold increase reduces the number of chloroplasts (Stokes *et al*., 2000), while loss-of-function mutants of *AtFtsZ2-1* show severe defects in chloroplast division, resulting in an increase in the size and a decrease in the number of chloroplasts in leaf mesophyll cells (McAndrew *et al*., 2008; Schmitz *et al*., 2009). The same interval also contains Zm00001eb348290 which encodes a protein containing a 2Fe–2S ferredoxin-type iron–sulfur-binding domain, particularly notable given the validated role of another gene, ISCA, associated with another GWAS hit from this same study which is involved in iron–sulfur binding. The interval on chromosome 3 associated with variation in ΦNPQ included 12 annotated genes. One of these, Zm00001eb140660 encoded an NAD(P)H-dependent oxidoreductase whose Arabidopsis ortholog, *AT3G2789*0, has been shown to play a role in regulating the production of reactive oxygen species (Biniek *et al*., 2017), a consequence of excess photosynthetic energy which is not safely dissipated via the NPQ pathway.

The observation that loss-of-function alleles in Arabidopsis exhibit phenotypes consistent with GWAS results in maize supports two assertions. Firstly, while maize and Arabidopsis belong to plant lineages separated by >100 million years of evolution, the core components and regulators of photosynthesis-related traits may be conserved. This is consistent with several other recent cross-species analyses of genes involved in photosynthetic trait variation (Sahay *et al*., 2023, 2024a) and stands in contrast to the significant divergence in the roles of genes involved in determining phenotypes such as flowering time or plant morphology. Secondly, controlling for multiple confounding factors not only substantially increased heritability but substantially improved outcomes from GWAS, resulting in both more and stronger GWAS hits. Collectively the results of this study provide reasons for optimism about the feasibility of studying the genetic determinants of variation in photosynthetic performance under field conditions and ultimately applying the insights gained to both engineering and breeding more photosynthetically productive crops.

## Supplementary Material

eraf198_Supplementary_Data

## Data Availability

Photosynthetic parameters measured as part of this study are provided as Supplementary Data File S1, and complete results from genome-wide association studies conducted here are provided as Supplementary Data File S2 at https://figshare.com/s/8af0d240a1f39e6b6885. The genetic markers used in this study were a subset from the file ‘WiDiv.vcf.gz’ hosted on the Dryad repository: https://doi.org/10.5061/dryad.bnzs7h4f1 (Grzybowski *et al*., 2023). Gene expression data employed in this study are publicly hosted on FigShare https://doi.org/10.6084/m9.figshare.24470758.v1 and have been previously described (Torres-Rodríguez *et al*., 2024). The code employed to conduct analyses and generate figures in this paper is deposited in an associated GitHub repository: https://github.com/waqarali1994/MultispeQ.git.

## Supplementary data

The following supplementary data are available at *JXB*  online.

Table S1. Range of values considered biologically plausible and retained for quantitative genetic analyses for each trait analyzed in this study.

Table S2. Primer sequences used for PCR to verify the Arabidopsis T-DNA insertional mutants.

Table S3. Percentage of total trait variance explained by different experimental or environmental factors.

Table S4. Heritability estimates reported by SpATS for individual photosynthetic traits with and without the inclusion of covariates.

Table S5. Positions and statistical support for genetic markers across GWAS conducted with different sets of BLUEs.

Table S6. Absolute gene distance from the genomic position of GWAS hits for mentioned traits.

Fig. S1. Field layout and fitted spatial trend across the field for ΦPSII, ΦNPQ, and relative chlorophyll.

Fig. S2. Distribution of raw data points collected using MultispeQ for each of the 14 traits employed in this study.

Fig. S3. Distribution of best unbiased linear estimates calculated after spatial correction for each of the 14 traits employed in this study.

Fig. S4. Relationships between light intensity and photosynthetic trait values.

Fig. S5. Light intensity values recorded as part of photosynthetic trait phenotyping efforts.

Fig. S6. Results of a genome-wide association study conducted using BLUEs calculated while correcting for device-to-device variation.

Fig. S7. Phenotypes of insertion alleles of Arabidopsis genes homologous to maize candidate genes identified via GWAS at flowering stage.

Fig. S8. Effect of correcting MultiSpeQ-measured NPQ_T_ using dark-adapted *F*_v_/*F*_m_.
