# Supplementary material for: Quantitative genetics of photosynthetic trait variation in maize
Source: J Exp Bot. 2025 May 14;76(14):4141–53. doi: 10.1093/jxb/eraf198 (PMC12448886; doi:10.1093/jxb/eraf198)
Supplement: eraf198_Supplementary_Data [file eraf198_supplementary_data.pdf]

**Supplementary Table S1** Range of values considered biological plausible and retained for quantitative genetic analyses for each trait analyzed in this study.

| **Traits** | **Lower Bound** | **Upper Bound** |
|---|---|---|
| $ECS_T$ | 0 | 0.02 |
| $gH^+$ | 0 | 500 |
| $vH^+$ | 0 | 0.3 |
| ΦPSII | 0.2 | 0.7 |
| ΦNPQ | 0 | 0.7 |
| ΦNO | 0 | 0.36 |
| qL | 0 | 0.8 |
| $NPQ_T$ | 0 | 7 |
| Fv'/Fm' | 0.35 | 0.85 |
| PSI-ac | 0 | 9 |
| PSI-opc | 0 | 1.5 |
| PSI-orc | -2 | 2 |
| PSI-oxc | -2 | 3 |
| Relative chlorophyll | 38 | 100 |

**Supplementary Table S2** Primer sequences used for PCR to verify the *Arabidopsis* T-DNA insertional mutants.

| **Primer** | **Left Primers (LP)** | **Right Primers (RP)** |
|---|---|---|
| SALK_007055 | 5'-aagaagccattaaggagctgc-3' | 5'-atcatctggtcttgcatttgc-3' |
| SALK_080503 | 5'-aattacatgactcgcgctctg-3' | 5'-ttgatcccaaatcatcaaacc-3' |
| SALK_047115 | 5'-aaacttgtttcatttaatttgggc-3' | 5'-aaaagtcaagaaatggtgggg-3' |
| pROK2- LBb1.3 | 5'-attttgccgatttcggaac-3' | N/A |

N/A, not applicable.

**Supplementary Table S3** Percentage of total trait variance explained by different experimental or environmental factors.

| **Trait** | **Light Intensity** | **Genotype** | **Row** | **Column** | **Day** | **Residual** |
|---|---|---|---|---|---|---|
| Relative Chlorophyll | 12% | 21% | 16% | 0% | 7% | 43% |
| $ECS_T$ | 9% | 4% | 5% | 0% | 8% | 74% |
| $gH^+$ | 28% | 6% | 4% | 0% | 9% | 54% |
| $vH^+$ | 39% | 2% | 5% | 0% | 7% | 47% |
| ΦPSII | 47% | 0% | 7% | 0% | 19% | 28% |
| ΦNPQ | 18% | 0% | 6% | 0% | 24% | 52% |
| ΦNO | 13% | 2% | 4% | 0% | 15% | 65% |
| qL | 0% | 3% | 5% | 0% | 3% | 90% |
| $NPQ_T$ | 6% | 2% | 5% | 0% | 19% | 68% |
| Fv’/Fm’ | 6% | 2% | 5% | 0% | 22% | 66% |
| PSI-ac | 0% | 6% | 2% | 0% | 1% | 90% |
| PSI-opc | 92% | 0% | 1% | 0% | 1% | 5% |
| PSI-orc | 0% | 0% | 3% | 0% | 0% | 97% |
| PSI-oxc | 0% | 0% | 1% | 0% | 1% | 97% |

**Supplementary Table S4** Heritability estimates reported by SpATS for individual photosynthetic traits with and without the inclusion of covariates.

| Trait | Pre-covariates | Post-covariates |
|---|---|---|
| Relative Chlorophyll | 0.67 | 0.67 |
| qL | 0.15 | 0.29 |
| PSI-ac | 0.26 | 0.25 |
| $vH^+$ | 0.08 | 0.44 |
| PSI-ac | 0.07 | 0.2 |
| Fv'/Fm' | 0.05 | 0.2 |
| ΦNO | 0.15 | 0.16 |
| $gH^+$ | 0.25 | 0.33 |
| ΦPSII | 0 | 0.32 |
| ΦNPQ | 0 | 0.23 |
| $ECS_T$ | 0.15 | 0.22 |
| PSI-oxc | 0.01 | 0 |
| PSI-orc | 0.02 | 0 |
| PSI-opc | 0.04 | 0.05 |

**Note: 1.** Heritability estimated by fitting genotype as a random effect to all individual MultiSpeq measurements in SpATS, including only 2D spatial correction but not additional covariates. **2.** Heritability estimated by fitting genotype as a random effect to all individual MultiSpeq measurements in SpATS using a model which included 2D spatial correction, light intensity and ambient temperature as fixed effects and day of collection as a random effect.

**Supplementary Table S5** Positions and statistical support for genetic markers across GWAS conducted with different sets of BLUEs.

| CHR | POS | Trait | RMIP (including ambient temperature) | RMIP (excluding ambient temperature) | RMIP (including device) |
|---|---|---|---|---|---|
| chr9 | 94775951 | ΦPSII | 0.36 | 0.11 | 0.2 |
| chr9 | 25683346 | ΦNPQ | 0.35 | 0.11 | 0.37 |
| chr10 | 83741616 | qL | 0.34 | 0.08 | 0.24 |
| chr8 | 102200025 | ΦPSII | 0.26 | 0.11 | 0.44 |
| chr3 | 149998190 | ΦNPQ | 0.22 | 0.2 | 0.19 |
| chr9 | 21755970 | Relative Chlorophyll | 0.22 | 0.11 | 0.02 |

**Supplementary Table S6** Absolute gene distance from genomic position of GWAS hit for mentioned traits

| Trait | Chromosome | GWAS hit (letter) | Position (bp) | Gene ID | Absolute Distance (bp) |
|---|---|---|---|---|---|
| ΦPSII | 9 | B | 94775951 | Zm00001eb386270 | 45291 |
| ΦNPQ | 9 | C | 25683346 | Zm00001eb378270 | 26156 |
| ΦNPQ | 9 | C | 25683346 | Zm00001eb378260 | 31509 |
| ΦNPQ | 9 | C | 25683346 | Zm00001eb378280 | 31652 |
| ΦNPQ | 9 | C | 25683346 | Zm00001eb378290 | 33146 |
| ΦNPQ | 9 | C | 25683346 | Zm00001eb378250 | 47333 |
| qL | 10 | D | 83741616 | Zm00001eb416530 | 4335 |
| ΦPSII | 8 | E | 102200025 | Zm00001eb348440 | 68788 |
| ΦPSII | 8 | E | 102200025 | Zm00001eb348430 | 72593 |
| ΦPSII | 8 | E | 102200025 | Zm00001eb348450 | 132721 |
| ΦPSII | 8 | E | 102200025 | Zm00001eb348460 | 211712 |
| ΦPSII | 8 | E | 102200025 | Zm00001eb348470 | 217333 |
| ΦPSII | 8 | E | 102200025 | Zm00001eb348420 | 248723 |
| ΦPSII | 8 | E | 102200025 | Zm00001eb348410 | 328013 |
| ΦPSII | 8 | E | 102200025 | Zm00001eb348480 | 351721 |
| ΦPSII | 8 | E | 102200025 | Zm00001eb348490 | 453725 |
| ΦPSII | 8 | E | 102200025 | Zm00001eb348400 | 635783 |
| ΦPSII | 8 | E | 102200025 | Zm00001eb348390 | 638900 |
| ΦPSII | 8 | E | 102200025 | Zm00001eb348380 | 779054 |
| ΦPSII | 8 | E | 102200025 | Zm00001eb348370 | 846743 |
| ΦPSII | 8 | E | 102200025 | Zm00001eb348360 | 883619 |
| ΦPSII | 8 | E | 102200025 | Zm00001eb348350 | 963800 |
| ΦPSII | 8 | E | 102200025 | Zm00001eb348340 | 1022418 |
| ΦPSII | 8 | E | 102200025 | Zm00001eb348330 | 1084898 |
| ΦPSII | 8 | E | 102200025 | Zm00001eb348320 | 1104540 |
| ΦPSII | 8 | E | 102200025 | Zm00001eb348310 | 1149274 |
| ΦPSII | 8 | E | 102200025 | Zm00001eb348300 | 1150256 |
| ΦPSII | 8 | E | 102200025 | Zm00001eb348290 | 1309784 |
| ΦPSII | 8 | E | 102200025 | Zm00001eb348280 | 1313247 |
| ΦNPQ | 3 | F | 149998190 | Zm00001eb140650 | 29114 |

| | | | | | |
|---|---|---|---|---|---|
| ΦNPQ | 3 | F | 149998190 | Zm00001eb140660 | 49594 |
| ΦNPQ | 3 | F | 149998190 | Zm00001eb140670 | 51703 |
| ΦNPQ | 3 | F | 149998190 | Zm00001eb140680 | 112975 |
| ΦNPQ | 3 | F | 149998190 | Zm00001eb140640 | 113061 |
| ΦNPQ | 3 | F | 149998190 | Zm00001eb140690 | 145631 |
| ΦNPQ | 3 | F | 149998190 | Zm00001eb140630 | 169806 |
| ΦNPQ | 3 | F | 149998190 | Zm00001eb140700 | 171996 |
| ΦNPQ | 3 | F | 149998190 | Zm00001eb140620 | 254225 |
| ΦNPQ | 3 | F | 149998190 | Zm00001eb140710 | 272049 |
| ΦNPQ | 3 | F | 149998190 | Zm00001eb140720 | 293418 |
| ΦNPQ | 3 | F | 149998190 | Zm00001eb140730 | 318418 |
| Relative Chlorophyll | 9 | G | 21755970 | Zm00001eb377130 | 16491 |
| Relative Chlorophyll | 9 | G | 21755970 | Zm00001eb377140 | 18165 |
| Relative Chlorophyll | 9 | G | 21755970 | Zm00001eb377150 | 21106 |
| Relative Chlorophyll | 9 | G | 21755970 | Zm00001eb377160 | 25003 |

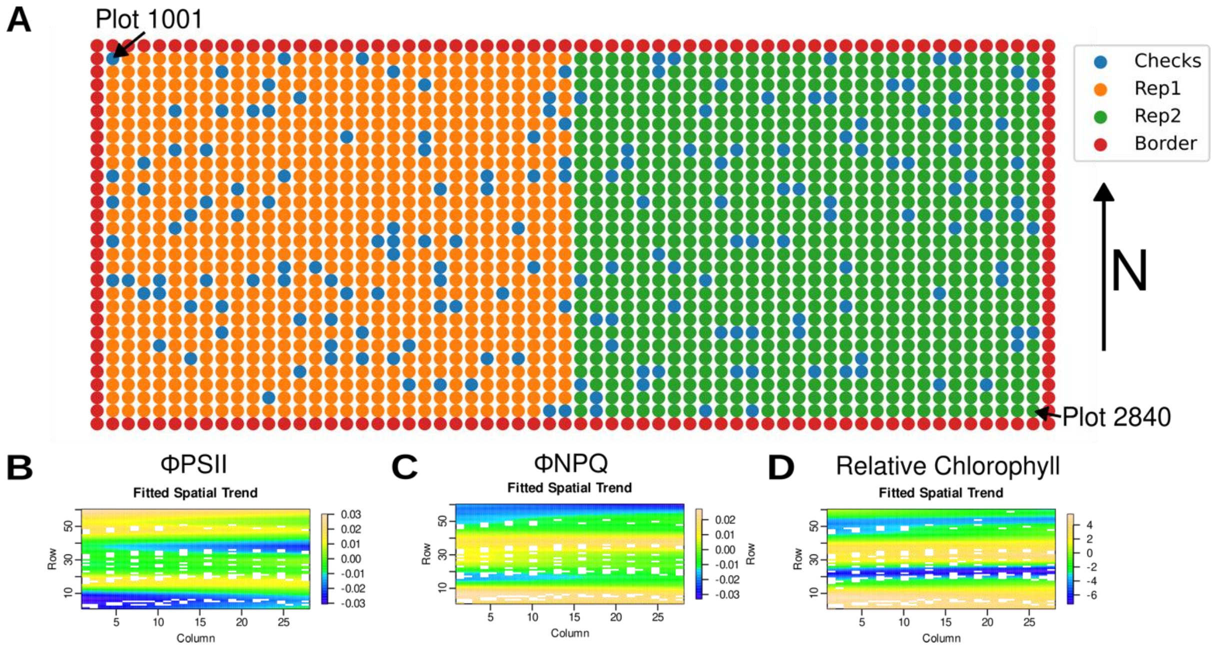


**Supplementary Fig S1 Field layout and fitted spatial trend across the field for ΦPSII, ΦNPQ and relative chlorophyll.** (A) Field layout representing the spatio-temporal arrangement of unique genotypes in two reps marked by green circles in rep1 and by orange circles in rep 2 along with blue circles representing the checks across both reps. (B) Spatial trend across the field (grid) created by SpATS in R. Color scale shows the range of ΦPSII values, blue color showing low values and yellow color showing higher ΦPSII values (C) Spatial trend across the field for ΦNPQ (D) Spatial trend across the field for relative chlorophyll

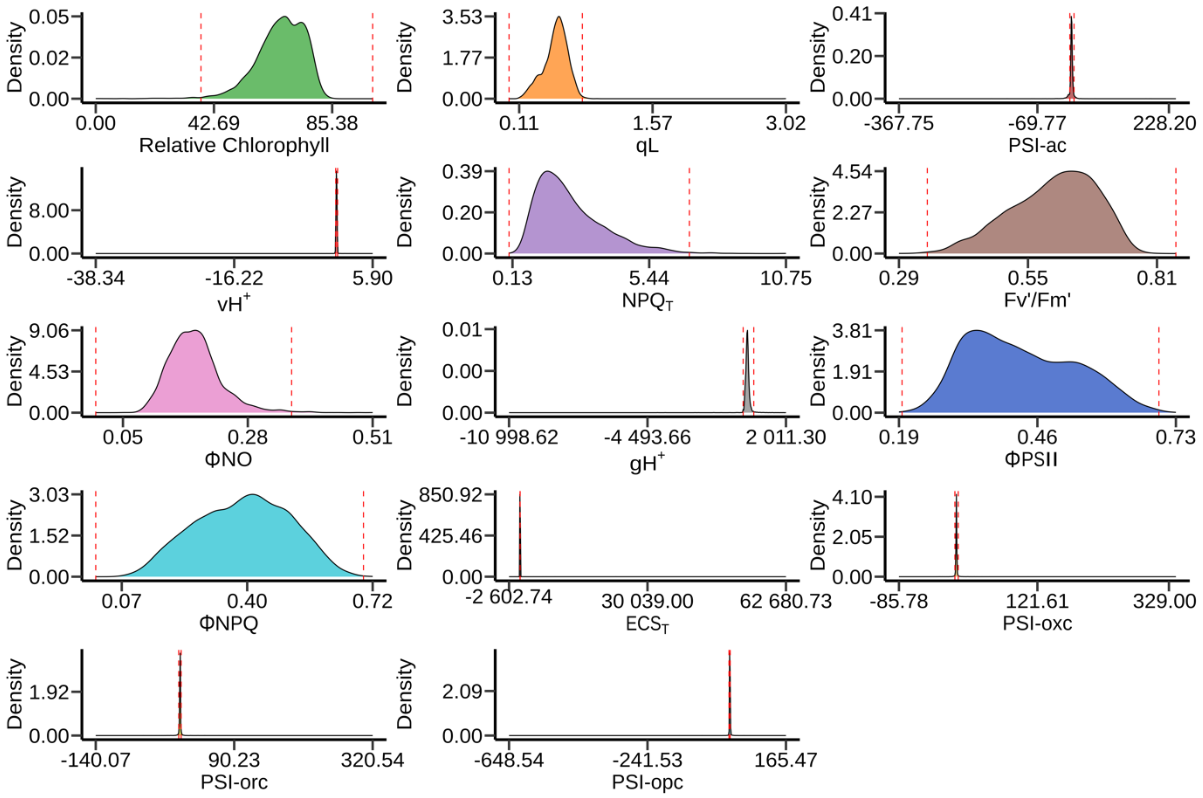


**Supplementary Fig S2 Distribution of raw data points collected using MultispeQ for each of the fourteen traits employed in this study.** Each panel shows a one-dimensional kernel density function fit to the raw data points without any outlier trimming and correction. The cutoffs are marked by red dotted placed vertically.

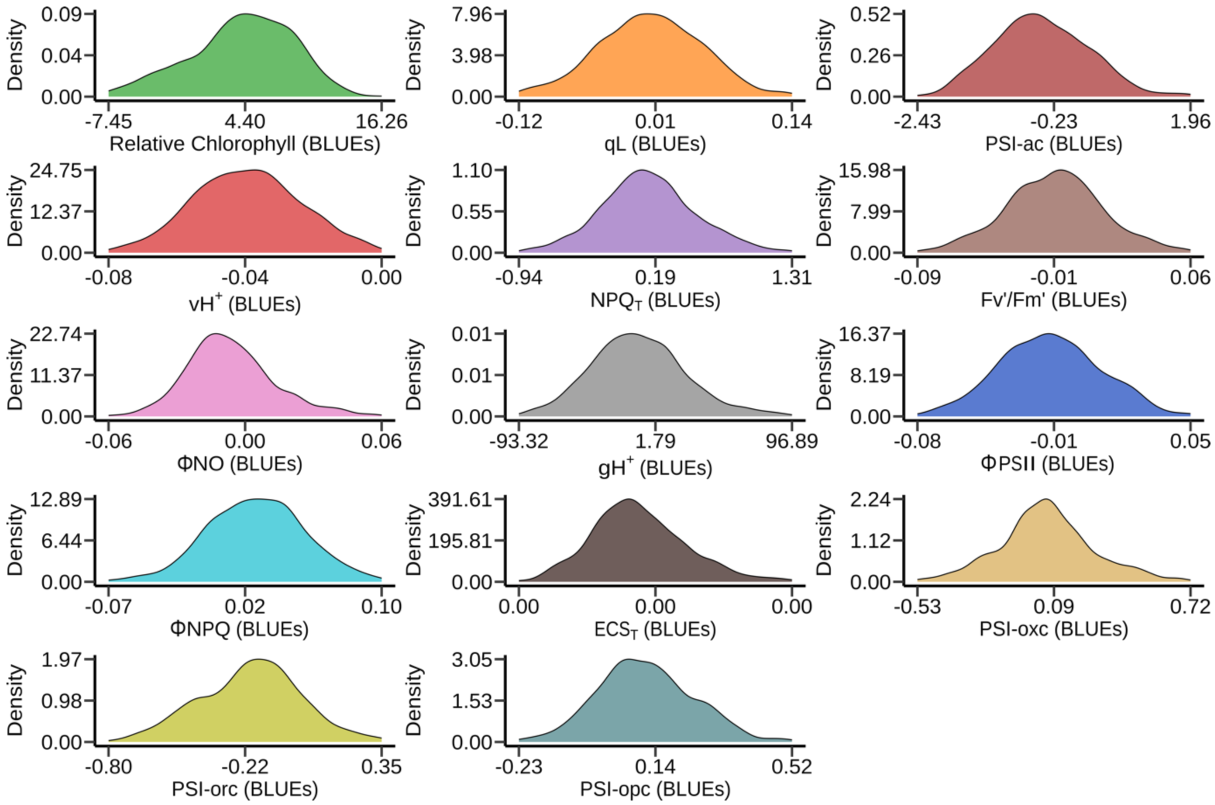


**Supplementary Fig S3 Distribution of best unbiased linear estimates calculated after spatial correction for each of the fourteen traits employed in this study.** Each panel shows a one-dimensional kernel density function fit to the best unbiased linear estimates output by the SpATS R package after outlier trimming and incorporating additional factors as described in methods.

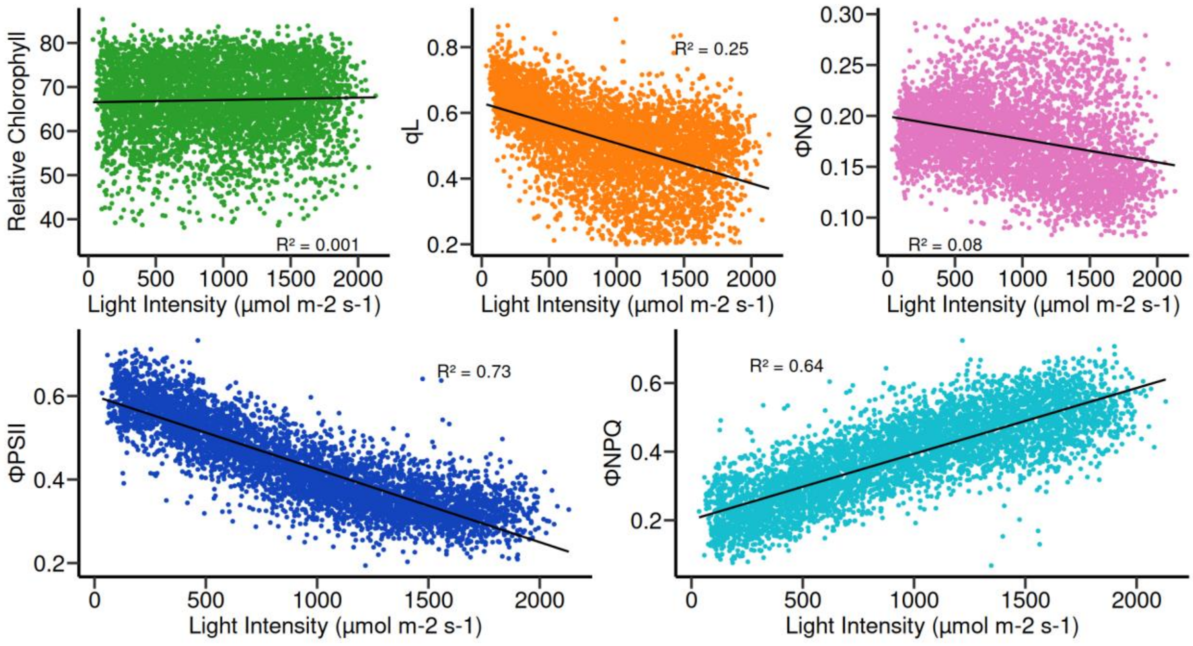


**Supplementary Fig S4 Relationships between light intensity and photosynthetic trait values.** Each panel displays the relationship between recorded trait values for each of the approximately 5,000 individual MuliSpeQ readings (data points) collected as part of this study and the light intensity value recorded by the same instrument at the same time. Solid black line indicates the best fitting linear relationship between light intensity and the trait of interest.

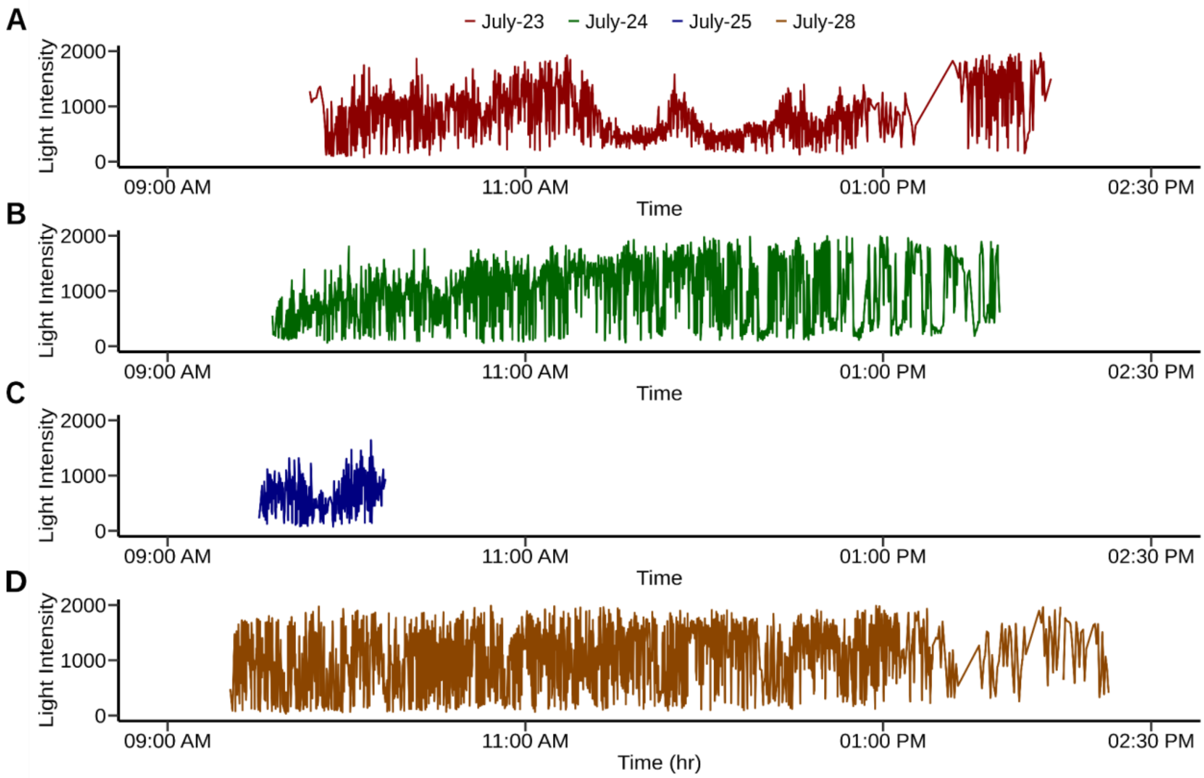


**Supplementary Fig S5 Light intensity values recorded as part of photosynthetic trait phenotyping efforts. (A)** Light intensity values recorded by six different MultiSpeQ instruments as part of 1,397 measurements collected on July 23rd, 2020. Light intensity quantified in units of micromoles per square meter per second (μmol m-2 s-1) (y-axis). The recorded time of collection for each individual measurement is indicated on the x-axis. **(B)** Light intensity values were recorded as part of 1,665 measurements collected on July 24th. **(C)** Light intensity values were recorded as part of 280 measurements collected on July 25th. **(D)** Light intensity values were recorded as part of 1,679 measurements collected on July 28th.

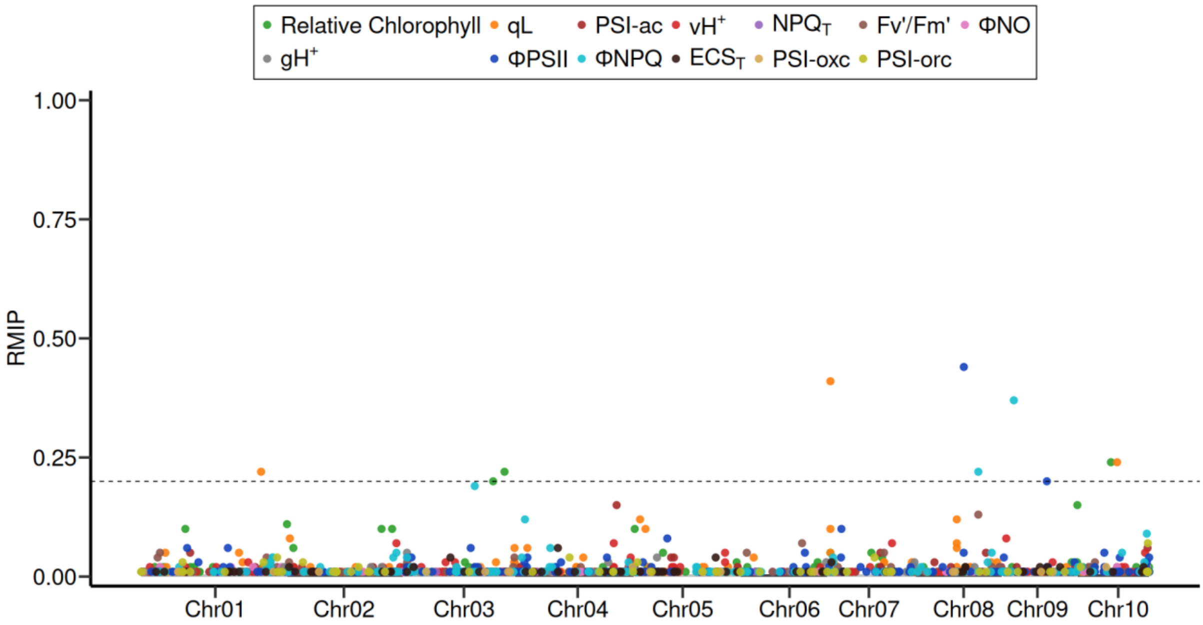


**Supplementary Fig S6 Results of a genome wide association study conducted using BLUEs calculated while correcting for device-to-device variation.** Statistical support and position on the maize genome for individual genetic markers associated with variation in BLUEs calculated using the model (Light Intensity + Ambient Temperature as fixed effects and Day and Device as random effects) using the SpATS package (1.0-18) implemented in R. Position on the x-axis indicates the physical position of the marker on the B73 RefGen V5 genome assembly. Position on the y-axis indicates the proportion of 100 iterations of FarmCPU GWAS where the marker was significantly associated with the given trait. Dashed line indicates a RMIP threshold of > 0.2.

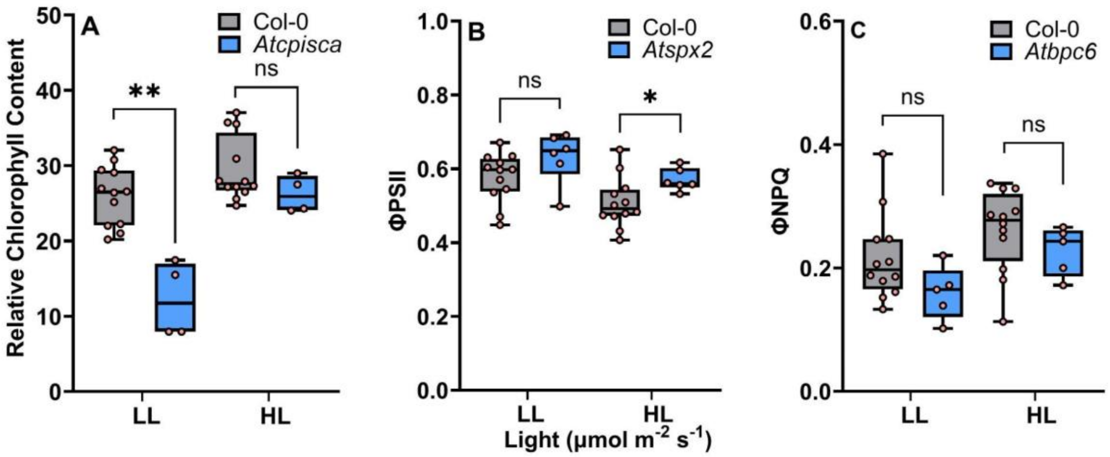


**Supplementary Fig S7 Phenotypes of insertion alleles of *Arabidopsis* genes homologous to maize candidate genes identified via GWAS at flowering stage.** Plants were grown under low light conditions (LL; 200 µmol $m^{-2}$ $s^{-1}$) and moved to high light conditions (HL; 550 µmol $m^{-2}$ $s^{-1}$) after six weeks and phenotypes were collected under both low light and high light after a 24-hour acclimation to higher light intensity. **(A)** Difference in relative chlorophyll content between *Atcpisca* mutant (*Chloroplast-localized ISCA protein*; SALK_007055; n=4) and wild type Columbia plants (CS6000; Col-0; n=12) for AT1G10500. (**B)** Difference in ΦPSII between *Atspx2* mutant (*SPX domain gene 2*; SALK_080503; n=7) and wild type Columbia plants (Col-0; n=12) for AT2G26660. **(C)** Differences in ΦNPQ between *Atbpc6* mutant (*Basic Pentacysteine 6*; SALK_0047115; n=5) and wild type Columbia plants (Col-0; n=12) for AT5G42520. $p^* \leq 0.05$, $p^{**} \leq 0.01$, $p^{***} \leq 0.001$ (unpaired, two-tailed t-test).

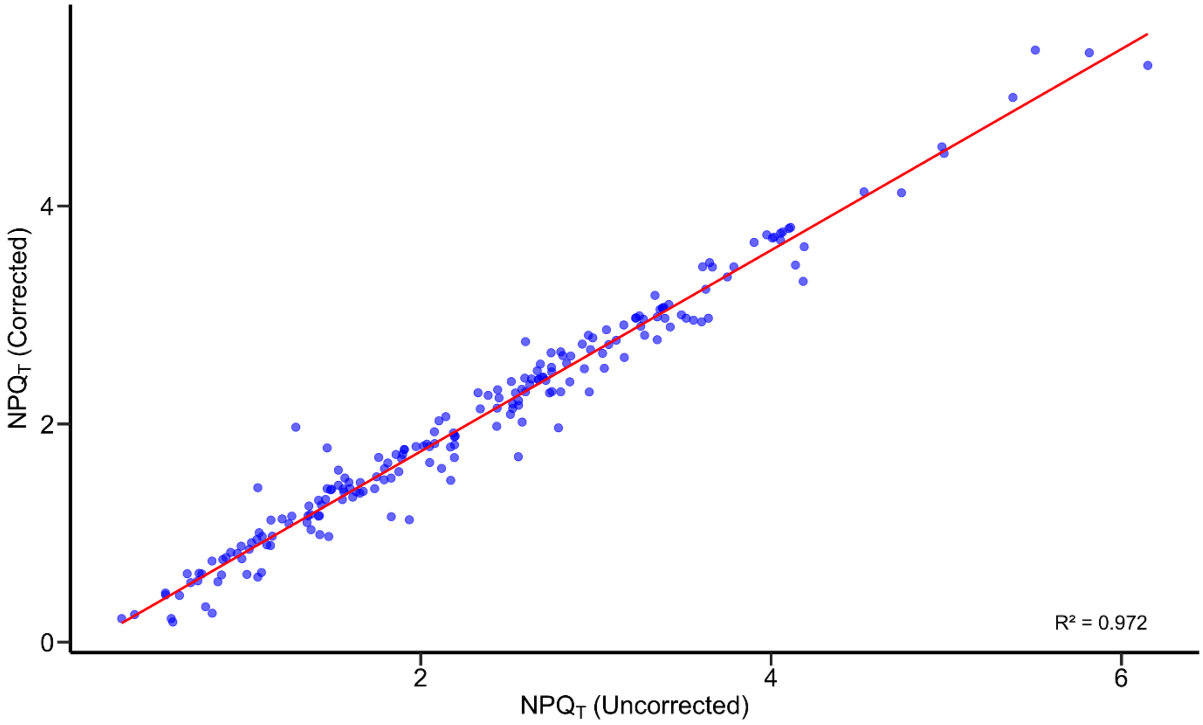


**Supplementary Fig S8.** Effect of correcting MultiSpeQ measured NPQ$_T$ using dark adapted $F_v/F_m$. Relationship between NPQ$_T$ values measured directly using the MultiSpeQ (x-axis) and NPQ$_T$ values calculated using dark adapted $F_v/F_m$ (y-axis). Each blue circle indicates one of 202 total measurements collected from a set of 20 maize genotypes collected across multiple time points. The data shown on the y-axis was previously published as part Sahay et al., 2024b.
